# Light-Dependent Control of Metabolism in Plants

**DOI:** 10.3390/ijms241813861

**Published:** 2023-09-08

**Authors:** Gábor Kocsy, Maria Müller

**Affiliations:** 1 Institute of Biology, Plant Sciences, NAWI Graz, University of Graz, 8010 Graz, Austria; 2 Agricultural Institute, Centre for Agricultural Research, ELKH, 2462 Martonvásár, Hungary

The energy of sunlight is converted into chemical energy during photosynthesis in plants. The blue and red spectral regimens are absorbed in the chloroplasts, and their energy ensures the production of NADPH and ATP by the use of water. Subsequently, atmospheric carbon dioxide is incorporated into ribulose 1,5-bisphosphate using NADPH and ATP, from which further carbohydrates will be synthesized. These carbohydrates will be used as the precursors of further compounds or for the production of energy for various metabolic processes during respiration. 

Besides ensuring energy for the various biochemical and physiological processes, light is also involved in the modulation of these processes. Plants have specific photoreceptors to perceive light signals from the sun including red- and far-red light-absorbing phytochromes (Phy), the blue and green light-perceiving cryptochromes (Cry), the blue light-sensing phototropins (PHOT), and the UV-B-absorbing UVR8 photoreceptor. These photoreceptors, in interaction with specific transcription factors, adjust the metabolic processes to daily and seasonal fluctuations of light intensity and spectrum.

Under high light intensity, the excess energy can lead to greater production of reactive oxygen species (ROS) in the chloroplasts, which results in oxidative stress. While a moderate increase in the ROS levels may activate various adaptive processes, their great production is toxic for the cells due to the damage of proteins, lipids, and nucleic acids. The amount of ROS is controlled by the redox system consisting of non-enzymatic (ascorbate, glutathione, tocopherol) and enzymatic (four enzymes of the ascorbate-glutathione cycle, superoxide dismutase, catalase, peroxidase) compounds. ROS and antioxidants regulate the redox environment in the cells, tissues, and organs of plants, which affects the activity of various proteins.

This Special Issue of the *International Journal of Molecular Sciences* entitled “Light-Dependent Control of Metabolism in Plants” consists of five original articles and one review. Although the influence of light conditions on metabolism has been studied earlier in several research groups, the valuable contributions of this issue give much new information in this field and indicate the unsolved problems worthy of future research.

Light can control metabolism at transcriptional, post-transcriptional, and post-translational levels. Eprintsev et al. [1] investigated the effect of light on the adenylate methylation of the promoter of the mitochondrial citrate synthase gene in maize leaves. This process regulates gene expression. While light and irradiation by red-light-activated DNA adenine methylase, darkness and far-red light inhibited it. A phytochrome B-associated control of the methylation of adenylate in the promoter of the citrate synthase gene was shown at all three GATC sites. The authors stated that the effect of phytochrome B on methylation is mediated by the phytochrome-interacting factor4. This is an important mechanism for the light-dependent control of the tricarboxylic acid cycle releasing the energy through the oxidation of acetyl-CoA derived from carbohydrates for the other metabolic processes. Since the blue light regimen is also absorbed by the photosynthetic pigments, it would be interesting to know whether it also influences the methylation of the citrate synthase gene. 

The light quality-dependent transcriptional control of basic metabolic processes such as nitrate, sulfate reduction, and maintenance of redox homeostasis was shown by Balogh et al. [2] in barley. The authors studied the effect of supplemental blue and far-red light on the proposed diurnal rhythm of the expression of genes related to these metabolic processes, which should be synchronized with photosynthesis providing energy and reducing power for them. After the maximum transcription of phytochrome- and cryptochrome- as well as circadian clock-associated genes during the light period, a great induction of the three investigated redox-responsive transcription factors was observed during the dark period. The peaking of the expression of the nitrate and sulfate reduction-, glutathione metabolism- and antioxidant enzyme-related genes occurred during the illumination of the plants. While blue light did not influence or inhibit the transcription of most of the genes, far-red light activated it. Based on these results, the authors created a model according to which far-red light plays a major role in the coordination of the sulfate and nitrate reduction and the redox system at the transcriptional level by the involvement of photoreceptors and the circadian clock. A continuation of this work could be the checking of the influence of light on the studied pathways at protein and metabolite levels.

The transcriptional control of the various biochemical processes by supplemental far-red light was also shown in Chinese kale in the work of Li et al. [3]. Far-red light induced the different expression of the genes associated with photosynthesis, hormone (auxins, gibberellins, cytokinins, jasmonate) biosynthesis, circadian rhythm, and signal transduction. Accordingly, the photosynthetic traits and hormone metabolism were modified by far-red light, too. These changes in turn resulted in the induction of the leaf growth (length, width, area) and flower budding rate, and subsequently in the increase in the weight of the edible parts. The transcriptional control of circadian rhythm and signal transduction by far-red light in Chinese kale corroborates the results obtained in barley [2]. The results demonstrated that modification of the light spectrum can increase the yield quality and quantity; therefore, the comparison of the effect of various spectral compositions on growth and development could be very useful during the cultivation of plants.

Supplemental UV-A light also modified the metabolism in kale grown under red and blue illumination as described by Jiang et al. [4]. It induced the accumulation of glucosinolates due to the activation of genes encoding transcription factors and enzymes associated with their synthesis. Glucosinolates are important secondary metabolites in the *Brassicaceae* family, and some of them have antioxidant functions, which may contribute to their efficient use against cancer. UV-A also increased the total flavonoid content, which could further increase the antioxidant capacity of this species. In addition, it also improved the growth of kale through the increase in chlorophyll content and its quality due to the elevation of protein and sugar levels. These observations give further evidence for the importance of the determination of optimal spectral conditions for indoor farming.

The modulation of metabolism by changing the light conditions could also be useful for the removal of pollutants from the environment as it was observed for nitrate in green *Microalgae* by Rani and Maróti [5]. Comparing various light intensities and spectral compositions, the greatest nitrate removal from wastewater was observed using a combination of blue and red light in high intensity. The effective nitrate removal was due to the activation of nitrate reductase at transcriptional and activity levels. In addition, genes encoding nitrate transporter also exhibited a greater induction under these light conditions. Thus, the determination of the appropriate light intensity and spectrum can greatly improve the phytoremediation capacity.

All five research articles demonstrated the transcriptional regulation of the metabolism by light and two of them [2,4] showed the involvement of the redox system in this process. Thus, the review of Gulyás et al. [6] about the light-dependent interactions between the redox system and miRNAs can be related to all of them since miRNAs can regulate the transcription of their target genes. Although none of the research articles presented the control of metabolism by miRNAs, this research field is worthy for future studies based on the review. The redox system plays an important mediator role in this process. Changes in light intensity and spectrum are perceived by the various photoreceptors, which can modulate the redox system and subsequently the redox-responsive miRNAs, transcription factors, and metabolism. There are interactions between the redox system and miRNAs since several miRNAs can regulate the level of various ROS and antioxidants. 

This Special Issue encompassed a very broad range of the light-dependent control of metabolism from the DNA-methylation through the transcription until the enzyme activation. It included both basic research questions (gene regulation) and applied research (waste management). Although this issue introduced several new aspects of the regulation of plant metabolism by intensity and spectrum of light, these investigations were carried out in various organs or tissues. The study of the metabolic changes at subcellular levels would be an interesting future research direction and it could contribute to the understanding of the fine-tuning of metabolism by light intensity and spectrum.
